# Capacitance Characteristics of Glass-Embedded Interdigitated Capacitors for Touch Sensing Applications

**DOI:** 10.3390/s25226941

**Published:** 2025-11-13

**Authors:** Apichart Kaewcharoen, Kirote Arpanutud, Prayoot Akkaraekthalin, Phongsaphak Sittimart, Suramate Chalermwisutkul

**Affiliations:** 1 High Frequency Systems Laboratory, King Mongkut’s University of Technology North Bangkok, Bangkok 10800, Thailand; s6109101960031@email.kmutnb.ac.th; 2 Thai Techno Glass Group PLC, Nakhon Pathom 73000, Thailand; 3 Faculty of Engineering at Sriracha, Kasetsart University Sriracha Campus, Chonburi 20230, Thailand; kirote.ar@ku.th; 4 Faculty of Engineering, King Mongkut’s University of Technology North Bangkok, Bangkok 10800, Thailand; prayoot.a@eng.kmutnb.ac.th; 5 Electrical and Computer Engineering Program, The Sirindhorn International Thai–German Graduate School of Engineering, King Mongkut’s University of Technology North Bangkok, Bangkok 10800, Thailand; phongsaphak.s@tggs.kmutnb.ac.th

**Keywords:** interdigitated capacitive sensor, glass-embedded structure, capacitance modeling, equivalent circuit modeling, touch sensing

## Abstract

**Highlights:**

**What are the main findings?**

- The capacitance behavior of interdigitated electrodes embedded between glass layers for touch sensing applications is accurately analyzed using equivalent circuit models for both touch and no-touch conditions. For a constant sensing area, wider electrode fingers reduce the number of fingers and the baseline capacitance in the no-touch state, resulting in improved touch sensitivity.
- Touch sensitivity—defined as the relative capacitance difference between touch and no-touch conditions—can be estimated during the electrode design stage by extracting the typical touch coupling capacitance under realistic application conditions. The dominant sensitivity factor is the coupling capacitance from the sensor electrodes through the fingertip and human body to ground, which leads to a lower capacitance measured across the positive and negative sensing electrodes under touch conditions.

**What is the implication of the main finding?**

- By analyzing and predicting the capacitance value during the sensor design stage, the electrode geometry can be tailored to match the desired sensitivity and measurement range of capacitive sensing chips.
- Tuning the sensitivity through electrode design and choice of overlay material provides an additional degree of freedom for system optimization, reducing dependence on the sensing chip’s intrinsic capability and threshold settings, and helping to avoid false alarms or missed detections.

**Abstract:**

This paper investigates the capacitance characteristics of a glass-embedded interdigitated capacitive sensor (IDCS) for touch-sensing applications. The study analyzes both baseline (no-touch) and touch-induced capacitance variations through a combination of analytical modeling and experimental validation. A multilayer analytical model is first employed to calculate the baseline capacitance of the proposed structure, followed by experimental measurements for model verification. Subsequently, an equivalent circuit model of the touched state is introduced to represent the interaction between the human fingertip, sensor electrodes, and earth-ground, explaining the observed capacitance reduction during a finger touch. Sensor prototypes with electrode finger widths of 1.4, 2.0, 2.4, and 3.0 mm were fabricated within a 40 × 40 mm^2^ sensing area. The baseline capacitance decreased from 28.6 pF at 1.4 mm to 12 pF at 3.0 mm electrode finger width, while the capacitance change upon touch ranged from 0.6–0.9 pF. Touch sensitivity for three test persons increased from about 1.7–4.6% at 1.4 mm to 5–7.6% at 3.0 mm electrode finger width. The results confirm that narrower-electrode designs yield higher absolute capacitance, whereas wider electrodes enhance touch sensitivity and provide greater uniformity within the defined sensing area. Overall, the findings validate the proposed IDCS configuration as a practical approach for realizing glass-integrated touch sensors and offer practical guidelines for optimizing electrode geometry in touch-based smart-glass applications.

## 1. Introduction

Capacitive sensing has become one of the most versatile and widely adopted technologies for human–machine interaction and environmental monitoring. Its use spans consumer electronics, automotive systems, healthcare, automation, and wearable devices, where robust, low-cost, and aesthetically pleasing interfaces are demanded [1,2,3,4,5,6]. Capacitive sensors provide numerous advantages over resistive or optical systems due to their long lifetime, immunity to mechanical wear, and compatibility with flexible or transparent substrates [7]. In recent years, capacitive technology has further expanded into emerging applications such as flexible electronic skin, smart packaging, and biomedical diagnostics [8,9,10]. Two major operational principles for such sensors are self-capacitance and mutual capacitance. Self-capacitive sensors measure the capacitance of an individual electrode with respect to ground, offering high sensitivity but limited multi-touch resolution. In contrast, mutual-capacitive sensors detect the coupling capacitance between transmitting and receiving electrode pair, allowing accurate localization and multi-point detection [11]. These developments highlight the adaptability of capacitive sensing to various applications [12,13].

Within this landscape, IDCS represent a particularly important configuration. Their planar geometry, consisting of interleaved electrode fingers, provides high sensitivity to dielectric changes in the surrounding medium, while also enabling compact and scalable layouts. IDCS have been applied across a wide range of domains: humidity and gas monitoring [14,15], bacterial detection and biosensing [16,17], microfluidics and liquid detection [18], soil moisture sensing in precision agriculture [19], and structural health monitoring of composites and cables [20,21]. Other studies have also demonstrated their use in biomedical applications, such as glucose monitoring and deformation detection in soft tissues [22,23,24]. This versatility stems from the strong interaction between fringing electric fields and the material under test, which makes IDCS particularly suitable for integration into layered substrates.

Glass is an attractive material for embedding interdigital capacitor (IDC) electrodes owing to its ubiquity, mechanical robustness, chemical stability, and transparency. The large surface area of glass panels allows broader sensing or interactive regions, making them suitable for touch-sensor integration in architectural environments. Previous studies [25] have reported that common architectural glasses exhibit relative permittivity of 6–9 with low dielectric losses, confirming their suitability as stable substrates for capacitive structures. Embedding electrodes within glass not only protects them from mechanical and environmental degradation but also aligns with the current trend of multifunctional “smart glass” materials, where windows and partitions serve as interactive interfaces. 

In capacitance modeling, analytical methods offer a low-cost and efficient approach compared with numerical or simulation-based techniques. However, in multilayer configurations, the calculation becomes complex due to field coupling across dielectric boundaries. Previous works [26,27] combined conformal mapping with the partial-capacitance method to evaluate IDC arrays embedded in multilayer dielectric stacks, assuming a monotonic permittivity profile. To overcome this limitation, an extended formulation based on the Schwarz–Christoffel conformal mapping and modified partial-capacitance network was proposed [28], enabling capacitance evaluation for multilayer structures with arbitrary dielectric variations. For the capacitance evaluation under touch conditions, the analysis is performed in terms of mutual capacitance [11], based on the equivalent-circuit representation. This approach allows the capacitance components to be considered in separate parts, reflecting the individual contributions from the sensor electrodes, coupling gaps, human body coupling, and earth-ground return path. Existing multi-layer IDCS models [26,27,28] are only applicable to uniform dielectric environments under no-touch conditions and cannot quantify the coupling effect between glass overlay thickness greater than 3 mm and human fingertip capacitance (1–5 pF). This forces the design of glass-based touch sensors to rely on extensive trial-and-error experiments, resulting in low design efficiency.

Building upon these theoretical foundations, this work aims to develop and validate a glass-embedded IDCS model for touch-sensing applications. The study combines analytical modeling, equivalent-circuit analysis, and experimental validation to investigate both the no-touch and touch capacitance behaviors. The proposed model enables quantification of capacitance variations associated with fingertip interaction, human body coupling, and grounding effects, providing a complete understanding of sensor performance. Unlike earlier models, this framework is the first to decompose human capacitance (*C_f_*) into fingertip–glass coupling capacitance (*C_t_*) and human body-to-ground capacitance (*C_h_*) and establishes a quantitative relationship between *C_f_* and electrode finger width (*F_w_*). It solves the core problem that traditional models cannot correlate electrode geometry with human-related factors. Thus, the proposed approach provides a unified analytical framework for IDC touch sensing, from the no-touch state with arbitrary geometry and overlay materials to the touched state with detailed charge distribution among all capacitive elements. The findings from this study are expected to establish fundamental design guidelines for integrating capacitive touch sensors into architectural glass systems.

## 2. Analytical Modelling of Interdigitated Capacitor Structures with Overlay Dielectric Layers

**Figure 1:**
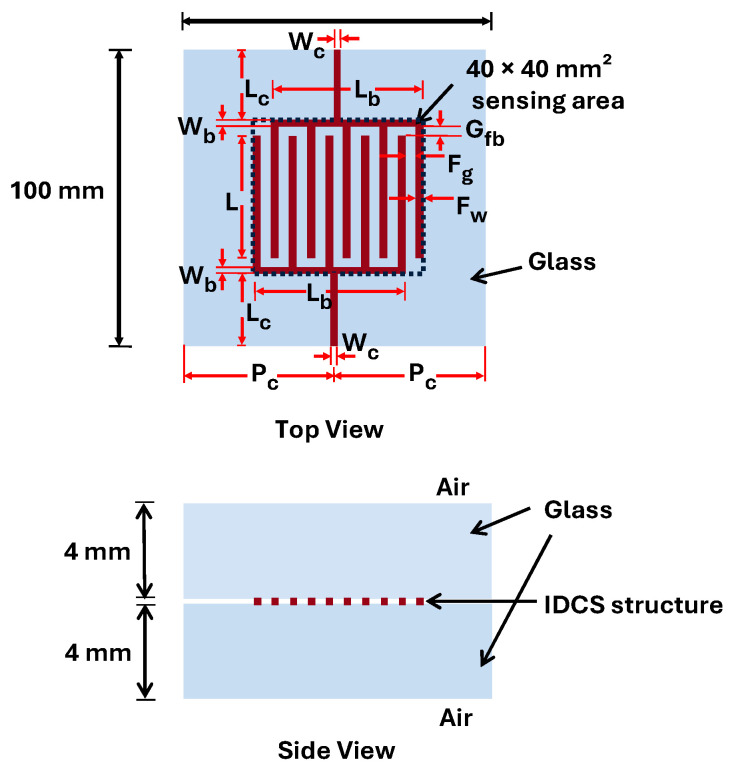
Proposed glass-embedded IDCS structure.

The proposed structure features IDC electrodes with an interlocking finger configuration embedded between two clear soda–lime glass layers, as illustrated in Figure 1. Typically, the soda–lime glass composes 70% SiO_2_, 15% Na_2_O (Soda) and 9% CaO (Lime) and other minor elements with the surface roughness of *Ra* = 1 nm. The dielectric constant of this glass is approximately 7.7 at 25 °C and increases to 8.3 and 11.1 at 150 °C and 250 °C, respectively [29]. Therefore, its variation within 10–60 °C is negligible. The electrodes were fabricated from an adhesive copper sheet with a total thickness of 0.05 mm, consisting of 0.02 mm copper and 0.03 mm acrylic-based adhesive. The sheet exhibits a surface resistance below 0.05 Ω/□ and has no surface treatment or anti-oxidation coating applied, ensuring sufficient conductivity while maintaining planar integration within the glass. Each electrode finger is defined by three geometric parameters: finger width *F_w_*, finger gap *F_g_*, and finger length *L*. The overall proposed electrode design provides an effective sensing area of 40 mm × 40 mm, which is deliberately larger than conventional fingertip-based touch sensors to accommodate broader interactive zones suitable for architectural applications. The encapsulating glass layers are rectangular sheets with dimensions of 100 mm × 100 mm and a thickness of 4 mm each, selected to represent realistic dimensions of residential glass installations. In this analysis, the relative permittivity of glass (*ε_glass_* = 7.7) and air (*ε_air_* = 1.0) are adopted as material parameters in the analytical model to evaluate the capacitance. The following section describes the analytical model used to determine the capacitance of the structure in the no-touch condition, and the equivalent electrical circuit employed to evaluate the capacitance during the touch condition.

### 2.1. Proposed IDCS Under the No-Touch Condition

#### 2.1.1. No-Touch Capacitance Calculation

The no-touch condition of the proposed glass-embedded IDCS structure refers to the state in which the capacitance characteristics depend solely on the intrinsic material composition of the structure. This condition is regarded as the baseline capacitance, determined by the dielectric layers consisting of glass encapsulation and surrounding air regions. The glass layers covering the sensing area are assumed to exhibit homogeneous and isotropic dielectric properties. For capacitance evaluation, the analytical approach proposed in [28] is employed to calculate the capacitance of the IDCS by considering the multilayer dielectric configuration with glass–air interface.

**Figure 2:**
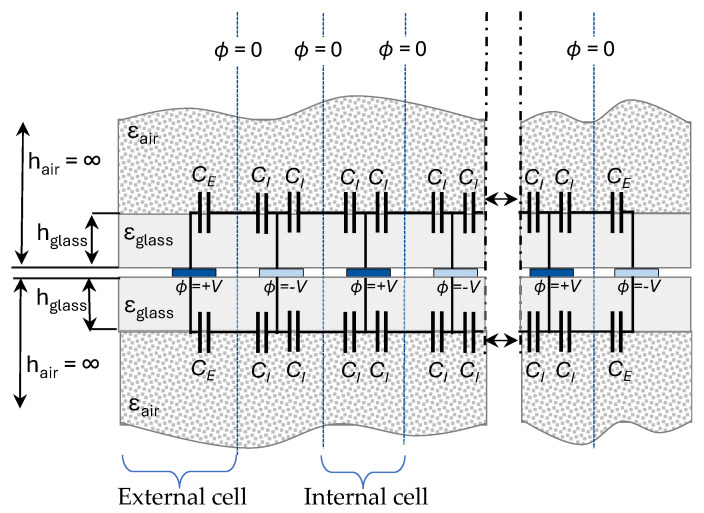
Equivalent circuit model of an IDCS structure embedded between two glass sheets.

From the proposed structure, the equivalent model of the IDCS embedded between two glass sheets is illustrated in Figure 2. Adjacent electrode fingers are applied with opposite potentials, establishing a virtual zero-potential electric wall between them. The capacitance between an inner electrode finger and this wall is called the inner-cell capacitance *C_I_*, while the capacitance between an outer electrode finger and the same wall is termed the external-cell capacitance *C_E_*. These two values form the foundation for computing the total capacitance of the structure.

In other multilayer configurations, dielectric constants of the layers can be arranged arbitrarily. However, in our case, the variation is distinctly monotonic since the structure consists of only two layers above and below the IDCS electrode—a finite glass layer and an infinite air layer. The height of each layer is referenced from the electrode surface, and an open-configuration model [28] is applied due to the infinite air layers.

The total capacitance of the IDCS is obtained by summing the contributions from the dielectric layers above and below the sensor. The analysis is applied separately to each half-space across the glass–air interface, and the resulting capacitances are combined to yield the overall no-touch capacitance *C_no-touch_* for *N* electrode fingers, as expressed by

(1) $$C_{\mathrm{no}\text{-}\mathrm{touch}\;}=2\;\cdot (\left(N-3\right)\frac{C_{I}}{2}+2\;\cdot \frac{C_{I\;}\;\cdot {\;C}_{E}}{C_{I\;}+C_{E}})$$

Based on the capacitance components in the equivalent circuit model, the calculation of these components is determined by two key non-dimensional parameters: the metallization ratio *η* and the height-to-width ratio r. These parameters are defined as follows:

(2) $$\eta \;=\;\;\frac{F_{w}}{F_{w}+F_{g}}$$

and

(3) $$r\;=\frac{h}{2\;\cdot \;\left(F_{w}+F_{g}\right)}$$

In this study, *F_w_* and *F_g_* were designed to be identical so that *η* = 0.5 to achieve a well-distributed sensing field across the region, minimizing areas with low field density. According to previous studies [26,27], an increase in r can cause the capacitance value to reach saturation beyond a certain point. In our configuration, r varies with *F_w_* and *F_g_* since the glass thickness is kept constant. For the infinite air layer, r approaches infinity with *h* → ∞. The design parameters *η* and *r* from Equations (2) and (3) are necessary for the calculation of the partial capacitance values *C_I_* and *C_E_* in Equation (1). The calculation procedure has been thoroughly elaborated in [26,27,28] starting from calculation of the cell constants $k_{cell\;}^{c}$ for the inner and external cells (see Figure 2) according to the calculation steps in Table 1 and Table 2 below. In our scenario, the overlay consists only of glass and air layers with monotonically decreasing dielectric constant away from the electrode plane.

The function *K*(*k*) in Table 1 and Table 2 represents the complete elliptic integral of the first kind. In case of the inner cells, $\vartheta_{2}(0,\;q)$ and $\vartheta_{3}(0,\;q)$ are the Jacobi theta functions of second and third type, respectively, and $sn\left(x,m\right)$ is the Jacobi elliptic sine function.

Finally, the partial capacitance values *C_I_* and *C_E_* must be calculated from *η* and *r* from Equations (2) and (3), the electrode finger length *L* and the cell constants $k_{cell\;}^{c}$ with

(4) $$C_{I}=\;L\cdot \;\left[(\varepsilon_{\mathrm{glass}}-\varepsilon_{\mathrm{air}}{)\;k}_{\mathrm{cell}}^{c}\left(\eta ,r_{\mathrm{glass}}\right)+\varepsilon_{\mathrm{air}}k_{\mathrm{cell}}^{c}\left(\eta ,\infty \right)\right]$$

(5) $$C_{E}=\;L\cdot \;\left[(\varepsilon_{\mathrm{glass}}{-\varepsilon_{\mathrm{air}})\;k}_{\mathrm{cell}}^{c}\left(\eta ,r_{\mathrm{glass}}\right)\;+\varepsilon_{\mathrm{air}}k_{\mathrm{cell}}^{c}\left(\eta ,\infty \right)\right]$$

#### 2.1.2. Experimental Validation of the No-Touch Capacitance Model

**Figure 3:**
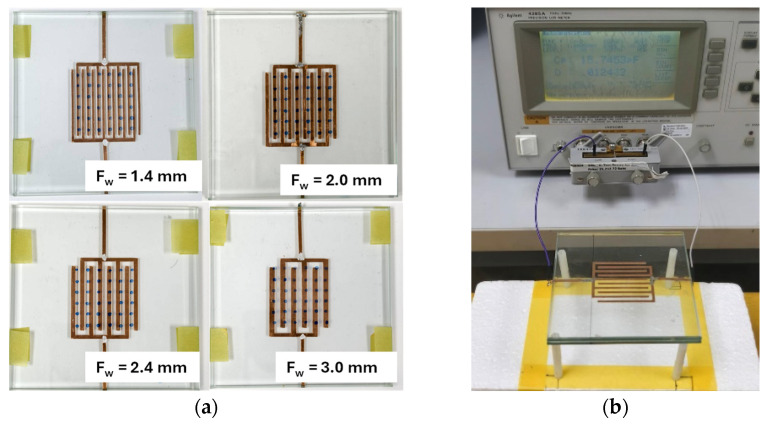
(**a**) Fabricated glass-embedded IDCS samples with different *F_w_* and (**b**) Experimental setup for capacitance measurement of the IDCS samples using Agilent^TM^ 4285A LCR meter.

Experimental investigation was conducted to validate the analytical model developed for predicting the no-touch capacitance of the proposed IDCS structure. The fabricated electrodes were designed by varying their geometrical parameters to evaluate the influence of electrode dimensions on the total capacitance. For the measurement setup, IDCS electrodes with different *F_w_* and *F_g_*, based on the parameters summarized in Table 3, were fabricated using copper foil with a thickness of 0.05 mm as shown in Figure 3a. These electrodes were adhered to the surface of a 4 mm clear glass plate measuring 100 mm × 100 mm and then sandwiched with another clear glass plate of the same size and thickness. Subsequently, two 200 mm long copper wires were connected to both sensor electrodes to facilitate capacitance measurement using Agilent^TM^ 4285A LCR meter (Agilent Technologies, Penang, Malaysia). The capacitance measurements were performed at a test frequency of 1 MHz with an AC drive level of 1 V and no DC bias applied to the device under test. The laboratory environment was controlled at a temperature of 25 ± 1 °C and an ambient humidity of 55 ± 1%. Measurements were conducted without electromagnetic shielding, and the ambient electric field strength was not measured due to the lack of suitable instrumentation.

The experimental setup in Figure 3b shows how the IDCS sample was connected to the test fixture of the LCR meter during capacitance measurement. This test setup was designed to minimize environmental interference and maintain measurement consistency. Prior to measurement, the LCR meter was calibrated at the ends of the connecting wires using short and open terminations. The capacitance was recorded 15 s after initiating the measurement to ensure a steady-state reading. After the waiting time, the value was stable with variation of only a few femtofarad corresponding to measurement uncertainty of less than 1%. The measured capacitance values were then compared with those predicted by the analytical model under the no-touch condition to assess the validity and accuracy of the model in representing the baseline capacitance behavior.

**Figure 4:**
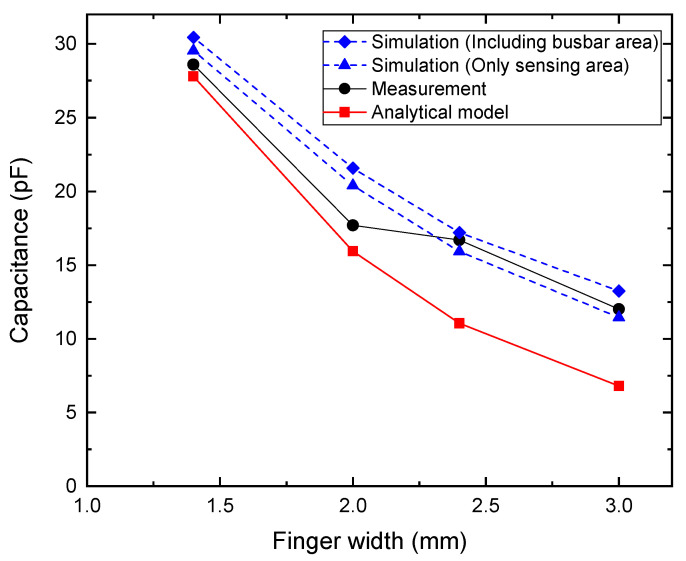
Comparison between simulation, analytical model predictions and experimental capacitance measurements in the no-touch condition.

**Figure 5:**
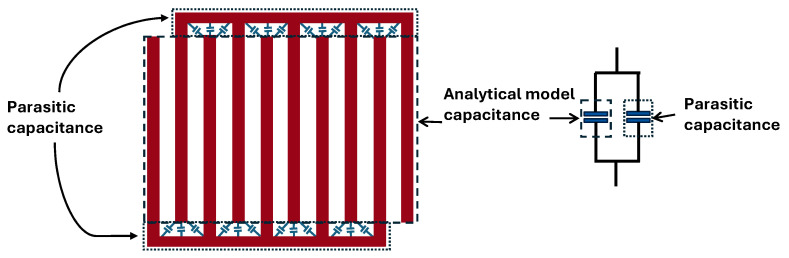
Equivalent circuit of the added parasitic capacitance at the electrode-finger ends not considered by the IDC analytical model.

Figure 4 shows the comparison between the capacitance values predicted by the analytical model and those obtained from measurements as a function of the *F_w_*. Both results exhibit a decreasing trend with increasing *F_w_*, confirming that the capacitance of the IDCS structure reduces as the electrodes become wider within a fixed sensing area due to decreased number of electrode fingers, reduced electrode length and wider electrodes’ gaps. At *F_w_* = 1.4 mm, the measured capacitance was approximately 28.6 pF, notably higher than the analytical prediction of approximately 27.8 pF. As *F_w_* increased to 2.0 mm, the analytical model estimated IDCS capacitance of 15.9 pF, whereas the measurement yielded a larger value of 17.7 pF. A similar tendency was observed at *F_w_* = 2.4 mm, in which the analytical model and the measured values were 11.0 pF and 16.7 pF, respectively. Finally, at *F_w_* = 3.0 mm, the results diverge more significantly, with the analytical model predicting 6.8 pF and the measurement indicating 12.0 pF. These results indicate that while the analytical model correctly captures the overall decreasing trend, it tends to underestimate capacitance across all *F_w_*, compared with the measured one. This deviation is primarily due to the simplifying assumptions inherent in the analytical approach, which does not fully capture parasitic effects and electrode edge conditions that occur in practical fabrication. As the electrode width increased, the difference between the two methods became more significant, particularly due to the parasitic capacitance between the busbar and the electrodes’ ends as depicted in Figure 5.

To verify this hypothesis, electromagnetic simulations using Ansys (Version 2024 R2) Q3D capacitance extractor were performed both with and without bus bars. By directly assigning potentials to the electrode fingers, the effect of the bus bars was excluded. Additional simulations, including the bus bars showed that they contribute to an increased total capacitance, with a stronger effect for wider electrode fingers. For narrow fingers, the analytical model (which neglects bus bars) agrees more closely with measurements, whereas for wider fingers, the simulation including bus bars provides a closer match as shown in Figure 4. Discrepancies may be attributed to simulation settings (e.g., mesh) and uncontrolled environmental factors. Table 4 presents the relative errors (%) of the analytical model and simulations with and without busbars, along with the percentage contribution of the busbars to the total capacitance.

### 2.2. Proposed IDCS Under the Touch Condition

#### 2.2.1. Touch Capacitance Model

**Figure 6:**
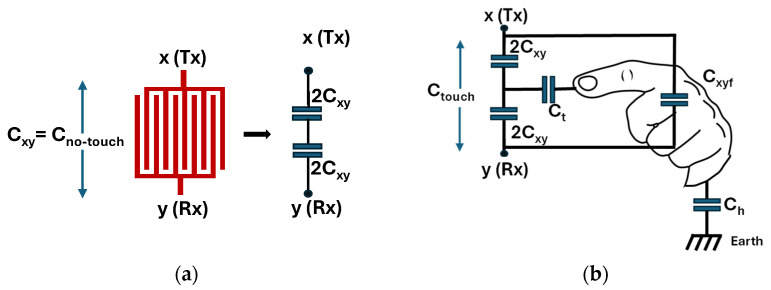
Capacitive touch sensor model under (**a**) no-touch and (**b**) touch condition.

By providing an electrical potential with opposite polarities to both electrodes of IDCS, electrode x with its positive and y with its negative potential can be referred to as the transmit (Tx) and receive (Rx) electrode, respectively (see Figure 6a). The IDCS no-touch capacitance, now referred to as C_xy_ can be represented by two series capacitors with a value of 2*C_xy_* as depicted in Figure 6a. For touch sensing applications, the IDCS in touched state cannot be modelled only by an additional coupling capacitance due to the permittivity of human fingertips. Instead, the finger touch also establishes a return-to-ground path over a capacitor that redistributes the fringing field between the transmit (x) and receive (y) electrodes, as depicted in Figure 6b.

**Figure 7:**
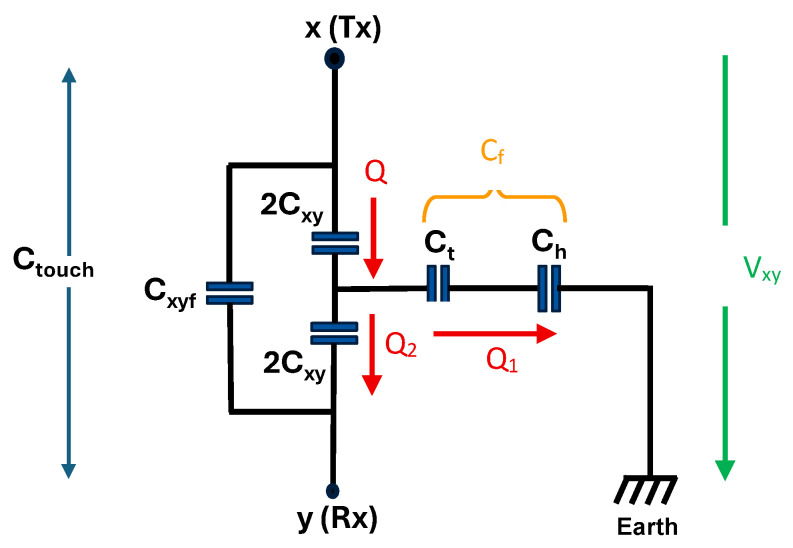
Charge distribution within the IDCS in touch state.

As a result, additional capacitance components are introduced into the system, including the touch capacitance *C_t_* between the fingertip and the virtual node between both electrodes in Figure 6b) through the glass overlay, the fingertip-to-ground capacitance *C_h_*, and the mutual coupling capacitance *C_xyf_* formed between the transmit and receive electrodes through the fingertip. These capacitances collectively modify the overall equivalent circuit of the structure, influencing both the charge distribution and the measured capacitance during the touch state. The capacitance change measured across x and y electrodes due to a touch event consists of two opposing effects. The mutual coupling capacitor *C_xyf_* through the fingertip tends to increase the measured capacitance between Tx and Rx electrodes. Simultaneously, the touch capacitance *C_t_* together with the human body capacitance *C_h_* establishes a return-to-ground path diminishing the net charge storage between x and y and thus reducing the x-y mutual capacitance. Charge distribution in the IDCS touch state is illustrated in Figure 7.

By considering the charge *Q* stored within the upper 2*C_xy_*, *Q* is the sum of charge *Q*_1_ stored in the ground return path over *C_t_* and *C_h_* and *Q*_2_ stored in the lower 2*C_xy_* so that,

(6) $$Q\;={\;Q}_{1}+{\;Q}_{2}$$

To simplify the mathematical expression, the total return path capacitance C_f_ resulting from the *C_t_* connected with *C_h_* in series is defined as

(7) $${\;C}_{f}\;=\frac{C_{t}C_{h}}{C_{t}+C_{h}}$$

Usually, the human body capacitance *C_h_* is large compared to the touch capacitance *C_t_* of the fingertip so that *C_t_* is the major element that influences the capacitance change and thus, the sensitivity of the touch sensor. By considering the node y (Rx) as a virtual ground, the lower 2*C_xy_* connected to the Rx electrode is in parallel with *C_f_*. The total capacitance between x and y without considering *C_xyf_* is calculated to

(8) $${\;C}_{touch}-{\;C}_{xyf}=\frac{2C_{xy}(2C_{xy}+C_{f})}{4C_{xy}+C_{f}}$$

With the voltage V_xy_ across the Tx and Rx electrodes, Q can be calculated with

(9) $$\;Q\;=\frac{2V_{xy}C_{xy}(2C_{xy}+C_{f})}{4C_{xy}+C_{f}}$$

According to the concept of a charge divider, charge *Q* in the upper 2*C_xy_* is divided into *Q*_1_ stored in *C_f_* and *Q*_2_ stored in the lower 2*C_xy_* so that

(10) $$Q_{1}:Q_{2}=C_{f}:{2C}_{xy}$$

Therefore,

(11) $$Q_{2}=\frac{4V_{xy}C_{xy}^{2}}{{4C}_{xy}+C_{f}}\;$$

The measured touch capacitance *C_touch_* consists of the contribution from *Q*_2_ from Equation (10) and the mutual coupling capacitance *C_xyf_* so that

(12) $$C_{\mathrm{touch}}=\frac{{{4C}_{\mathrm{xy}}}^{2}}{\;{4C}_{\mathrm{xy}}+C_{f}}+{\;C}_{\mathrm{xyf}}$$

#### 2.2.2. Effect of C_xyf_ on Measured Touch Capacitance

To analyze the impact of *C_xyf_* on the measured *C_touch_* value, the interaction between IDCS structure and a dielectric material on the glass surface above the sensing area is investigated. For this analysis, the influence of earth ground return path over *C_f_* is excluded. Therefore, Equation (12) is simplified by omitting *C_f_*, focusing solely on the direct electric field coupling between the sensor electrodes through the contacting dielectric material. To model the fingertip in our capacitance analysis, we approximated it as a cylindrical volume of water with a relative permittivity of approximately 78. In reality, the dielectric constant of human skin is lower due to its lower water content, ranging from about 20% at the surface to 60–70% within 0.1–0.2 mm. At 300 MHz, the average dielectric constant of hand tissue is reported to be ~44.3 with a conductivity of 0.49 S/m [30]. Therefore, the actual fingertip capacitance coupled to the electrodes is expected to be lower than that predicted by the water-cylinder model. This indicates that if the coupling capacitance is negligible in the 10–20 mm diameter water model, it will be even smaller for a real fingertip, supporting the assumption that its contribution can be considered negligible.

**Figure 8:**
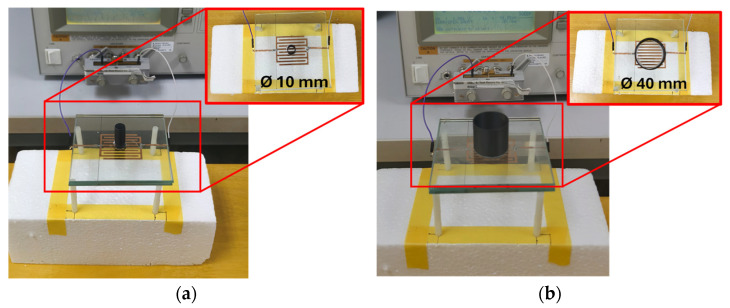
Measurement setup for C_xyf_ extraction using a water-filled cylindrical container with (**a**) 10 mm diameter (mimicking small fingertip touch) and (**b**) 40 mm diameter (mimicking palm touch).

An experimental setup was designed to evaluate the effect of *C_xyf_* using distilled water contained in a 3D-printed cylindrical structure with a height of 35 mm. The material used to fabricate the cylinder is ploy lactic acid (PLA). The wall thickness of the cylinder is 0.5 mm. The cylinder was positioned at the center of four sensor configurations with *F_w_* of 1.4, 2.0, 2.4, and 3.0 mm to simulate the electric field coupling effect of a fingertip in contact with the glass surface. Cylinders with diameters of 10, 20, 30, and 40 mm were used to represent various fingertip sizes. Figure 8 shows photographs of the experimental setup for *C_xyf_* extraction with different fingertip sizes.

Table 5 presents the measured capacitance change equivalent to *C_xyf_* resulting from filling the cylindrical structure with water. *C_xyf_* was calculated using Equation (12) while *C_f_* = 0 was assumed due to no ground return path. Placing an empty cylindrical container of any size on the glass surface above the IDCS sensing area did not change the measured capacitance, indicating that the electric field disturbance caused by the container is negligible for all IDCS samples. Filling the cylindrical structure with water caused a slight increase in capacitance, which became more pronounced with larger cylinder diameters and wider electrode fingers. This behavior results from the high permittivity of water influencing the measured capacitance. The extracted *C_xyf_* representing the electric field coupling through the dielectric material placed on the glass surface, was negligible for smaller cylinder diameters (10 mm and 20 mm). However, as the cylinder diameter or *F_w_* increased, *C_xyf_* became more significant, particularly for larger cylinder diameters of 30 mm and 40 mm. This trend indicates that the measured capacitance is directly governed by the contact area between the glass overlay of the sensor and water, highlighting the combined influence of contact area and electrode finger width on the measured capacitance.

In reality, the dielectric constant of human fingertips is lower than that of water (~78), with a reported value of around 60 [31]. Therefore, the actual fingertip capacitance coupled with the electrodes is lower than predicted by the water-cylinder model. For typical fingertip contact diameters of 8–10 mm, the coupling capacitance is negligible, causing minimal change in the total capacitance. Consequently, *C_xyf_* can be omitted from subsequent capacitance calculations.

To investigate the effect of overlay thickness, the glass above the IDC structure was varied from 2 mm to 4 mm. *C_xyf_* was extracted from Equation (12) for all glass thicknesses and water container diameters, with *F_w_* fixed at 2 mm. The results are listed in Table 6. It is notable that for the 3 mm glass overlay with a 40 mm diameter, *C_xyf_* is substantially larger than in other cases. This indicates that *C_xyf_* may no longer be negligible when the overlay is thin, the touch area is large, and the electrode fingers are wide.

#### 2.2.3. Effect of C_f_ on Touch Capacitance

According to the results of study regarding *C_xyf_* in Section 2.2.2, the variation of measured capacitance due to a touch event due to *C_xyf_* is negligible. Besides *C_xyf_*, the measured touch capacitance *C_touch_* is influenced by the touch return path capacitance C_f_ over the fingertip and human body. The return path capacitance *C_f_* vary from person to person and is dependent on several environmental factors. Thus, it is more practical to extract its typical values from experiments than predicting it using theoretical analysis or simulations. From Equation (12), *C_f_* can be expressed as

(13) $$C_{f}=\left(\frac{4C_{\mathrm{xy}}^{2}}{C_{\mathrm{touch}}-C_{\mathrm{xyf}}}\right)-4C_{\mathrm{xy}}$$

**Figure 9:**
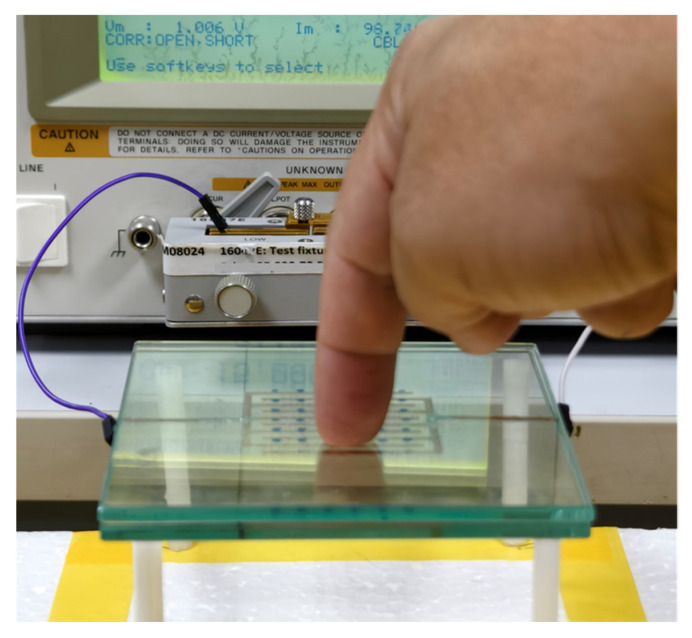
Capacitance measurement of the proposed IDCS in touch state with fingertip contact.

To analyze the capacitance change during a touch event, an experiment was conducted using IDCS with a 4 mm thick glass overlay as shown in Figure 9 for all *F_w_* values. Three healthy adult participants—test person 1 (male, 42 years), test person 2 (female, 33 years), and test person 3 (female, 38 years)—were involved in the test, each touching the center of the sensor with their index fingertip. Measurements were conducted after a typical workday (9 a.m.–5 p.m.) in a university office environment. No hand cream or other substances were applied before testing. Although individual skin humidity was not measured, the laboratory humidity sensor indicated an ambient humidity of 55%. Each participant was trained to apply a standardized touch force of 50 ± 5 g, verified using a digital weighing scale.

The following conditions were applied during the experiment:

- Footwear: Sports shoes (sneakers) with a 20 mm Ethylene Vinyl Acetate (EVA) foam cushion.
- Flooring material: 1.5 mm vinyl tile on a concrete floor.
- Ground reference of the instrument: The instrument was powered through a grounded building outlet, with its ground terminal connected directly to the earth ground.
- The participants were not galvanically connected to the instrument ground.

The IDCS capacitance was measured in touch and no-touch states using an LCR meter. From the measured data, *C_f_* was subsequently extracted using Equation (13).

Table 7 summarizes the measured IDCS capacitance values before and after fingertip contact, together with the extracted $C_{f}$ obtained from Equation (13) for two individuals and all $F_{w}$ values. For both individuals, the measured touch capacitance $C_{\mathrm{touch}}$ decreased during finger contact. As shown in Table 5, the extracted $C_{f}$ values for test person 2 are consistently higher than those for test person 1 across all $F_{w}$, indicating stronger coupling through the ground return path. This enhanced coupling between the IDCS and earth ground via the human body results in a greater reduction of capacitance during touch compared with the no-touch state. The variation in $C_{f}$ between individuals, likely due to anatomical differences such as fingertip size, may enable estimation of fingertip dimensions that is an aspect to be investigated in future work.

## 3. Touch Sensitivity

### 3.1. Sensitivity Calculation

Touch sensitivity was evaluated and compared for all cases listed in Table 7. Sensitivity was defined as the relative decrease in measured IDCS capacitance upon touch, calculated as

(14) $$\mathrm{Sensitivity}\;\mathrm{in}\;\%=\left(\frac{C_{xy}-C_{\mathrm{touch}}}{C_{\mathrm{xy}}}\right)\;\times \;100\%$$

**Figure 10:**
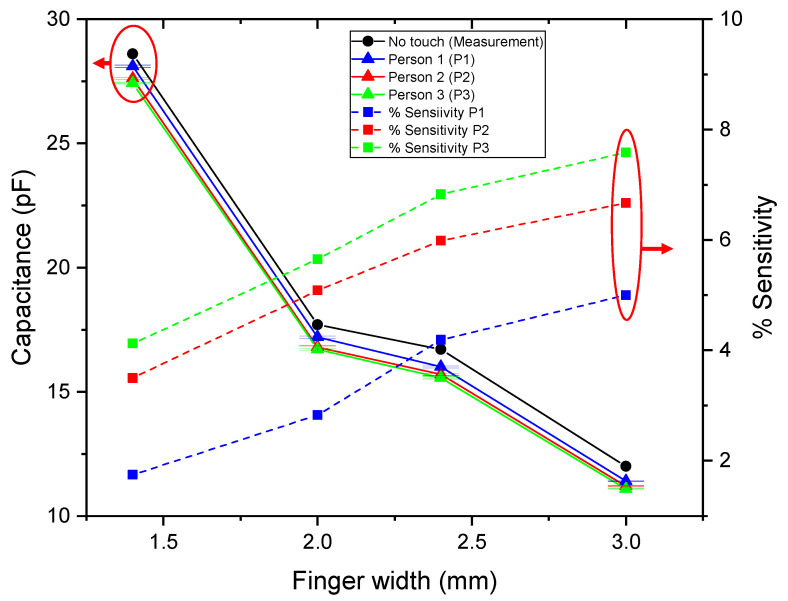
Measured capacitance and sensitivity of the proposed IDCS under fingertip touch with three participants across various electrode finger widths.

Measured capacitance values in touch and no-touch states for all *F_w_* and test individuals are plotted in Figure 9. The capacitance of the touch state of test person 1 is larger than that of test person 2 for all electrode widths indicating higher touch sensitivity of test person 2 compared to test person 1. Touch sensitivity from Equation (14) in case of test person 1 and 2 is plotted in Figure 9. It is observed that the sensitivity is increased by increasing *F_w_*. Considering the measured data from Table 7, the total capacitance reduction due to touch event is not significantly different considering all electrode width. However, large *F_w_* leads to reduced baseline capacitance of the no-touch state so that the relative reduction in % of the capacitance—the sensitivity—is higher in case of wide electrode finger. According to results in Figure 10 sensitivity at *F_w_* = 1.4 mm was lowest at 1.75% for test person 1 and 3.50% for test person 2, reflecting that the no-touch capacitance was large and the relative change compared with baseline was modest. With increasing *F_w_*, the sensitivity increased steadily, reaching 5.0% for test person 1 and 6.67% for test person 2 at *F_w_* = 3.0 mm.

### 3.2. Sensitivity Distribution over the Sensing Area

**Figure 11:**
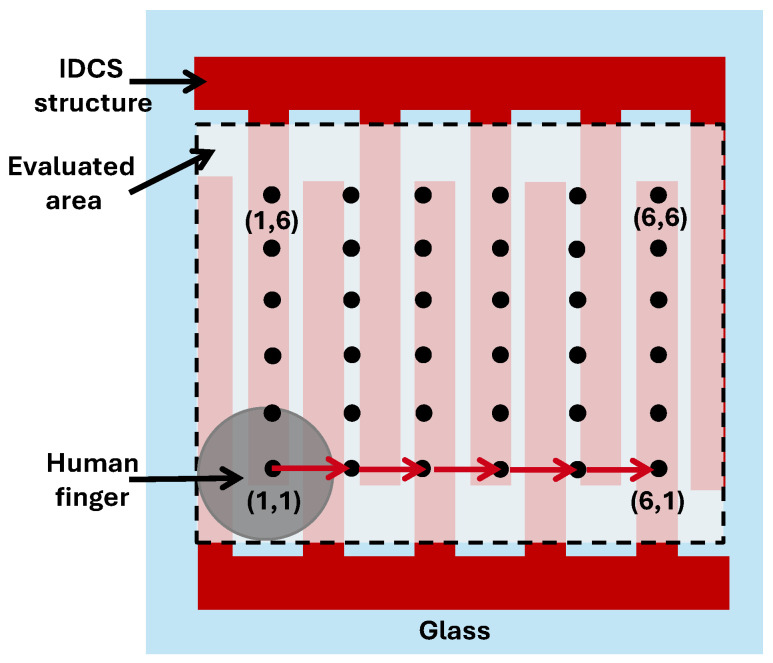
Drawing of proposed IDCS structure with 36 pixels (6 × 6 touch points) used for the sensitivity evaluation of the touch sensor.

Sensitivity distribution of the proposed structure was analyzed by defining 36 touch points within the sensing area of 40 × 40 mm^2^ on the glass overlay of the IDCS as illustrated in Figure 11. A finger touch experiment was conducted for all 36 touch points to evaluate the sensitivity distribution on the glass embedded IDCS. Touch sensitivity according to Equation (14) was calculated from the measured baseline and the touch capacitance.

**Figure 12:**
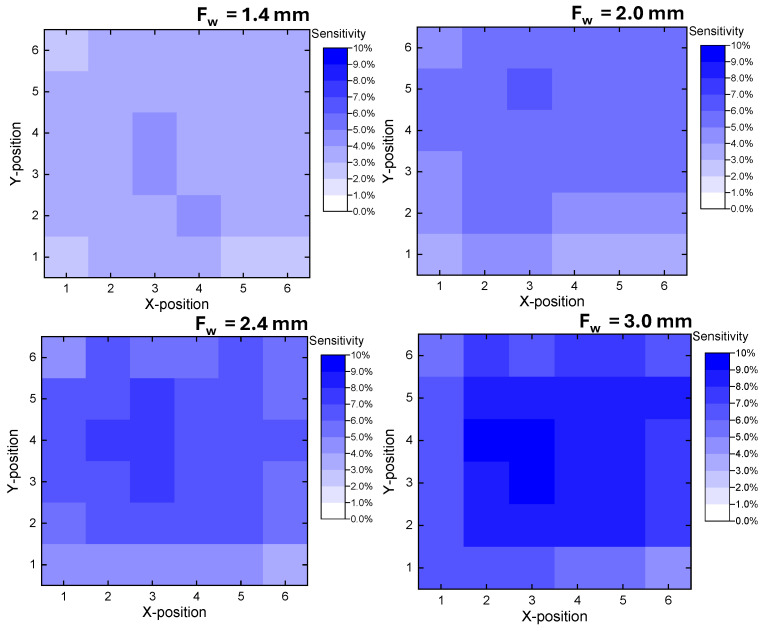
Touch sensitivity distribution over the sensing area of the proposed IDCS with various *F_w_*.

According to Figure 12, the sensitivity distribution exhibited distinct variations across 36 touch points within the sensing area. Overall, the sensitivity was relatively consistent across all touch points for all *F_w_*. For *F_w_* = 1.4 mm, the overall sensitivity was the lowest ranging from 2.3 to 3.9%. As the finger width increased to 2.0 mm and 2.4 mm, the sensitivity gradually improved, ranging from 3.5–5.9% and 4.4–7.3%, respectively. The 3.0 mm configuration provided the highest response, with sensitivities spanning 4.5–9.4%. These results demonstrate that the proposed structure maintains uniform touch detection performance within the defined sensing area across all *F_w_*. Despite the differences in magnitude, all cases exhibited a similar trend. Higher sensitivity was consistently observed in the central region of the structure, while the outermost areas, corresponding to the electrode edges, showed slightly lower sensitivity. This can be attributed to the weaker electric-field intensity near the periphery, resulting in reduced capacitive coupling with the fingertip compared to the central region where the field lines are more concentrated. Within the scope of this study, the uniformity of the sensitivity was found to be sufficient for reliable detection of human fingertip contact across the defined sensing area.

## 4. Discussion

This work investigates the capacitance characteristics of an embedded IDCS in both touch and no-touch states, focusing on the influence of sensor geometry and the dielectric properties of the overlay material, which in this study is glass. The analysis began with the baseline capacitance in the no-touch state. Experimental validation confirmed that the proposed analytical model can predict capacitance values, although measurements tend to be higher especially with relatively wide electrode fingers due to peripheral structures such as electrode interconnections and lead wires, which contribute parasitic capacitance. Despite this, the model effectively estimates the baseline capacitance range and offers flexibility for designing IDCS structures with arbitrary sensing areas, electrode geometries, and overlay properties. Thus, our study provides a versatile design framework applicable to touch sensors with single- or multilayer dielectric overlays of any permittivity.

In the touch state, contact on the dielectric surface introduces a coupling path from the virtual node between the electrodes to ground via the return-path capacitor *C_f_*, which primarily determines touch sensitivity. *C_f_* depends on several factors, including fingertip size, overlay thickness, and environmental conditions. Typically, *C_f_* is dominated by the smallest capacitor in the return path—the electrode-to-fingertip coupling *C_t_*. *C_t_* increases with two-finger touch, thinner overlays, or higher-permittivity materials. With an overlay separating the fingertip from the electrodes, *C_t_* reduces the measured touch capacitance. Another capacitance, *C_xyf_*, arises from inter-electrode coupling through the fingertip, increasing total capacitance, but its influence is smaller than *C_f_* under typical building glass thicknesses. Conversely, if the fingertip directly contacts the electrodes or if the overlay is very thin with wide electrode fingers, *C_xyf_* can dominate, resulting in an increase in total capacitance, similar to touchscreen or wearable sensors with thin overlays.

A review of recent literature on glass-embedded touch sensors shows that most studies focus on touchscreens or wearable devices with thin overlays, where touch increases capacitance due to *C_xyf_* [32,33]. In contrast, our glass-embedded sensor exhibits decreased capacitance under touch, highlighting a distinct sensing mechanism and design approach. The proposed structure offers key innovations, including predictable sensitivity through electrode and overlay design, compatibility with typical architectural glass thicknesses, and a practical design framework for integration into smart surfaces and interactive building elements. These features provide designers with clear guidance on expected capacitance trends and touch performance, demonstrating the novelty and practical relevance of our work.

## 5. Conclusions

This study proposed a glass-embedded interdigital capacitive sensor structure for touch-sensing applications. The proposed structure was analyzed using an analytical model to determine the baseline no-touch capacitance, which was subsequently validated through experimental measurements. The results showed good agreement between the analytical and measured capacitance values. As the electrode finger width increased, edge effects became more pronounced, causing the measured capacitance to notably exceed the analytical predictions. It was observed that electrode geometry, specifically finger width, length and gap, plays a decisive role in determining the total capacitance of the structure. For a defined sensing area, narrower electrode fingers with a larger number of fingers yielded higher baseline capacitance due to stronger fringing-field concentration, whereas wider electrode fingers with fewer fingers exhibited lower baseline capacitance.

Furthermore, a touch-capacitance model was introduced using an equivalent-circuit representation to describe the interaction between the fingertip and the sensor structure. During contact, a portion of the displacement charge is diverted through the fingertip and human body to the earth ground, forming a return path that reduces the measured mutual capacitance between the electrodes. Consequently, the total capacitance after touch decreases compared with the no-touch condition. Touch sensitivity was defined as the relative change in the measured IDCS capacitance, expressed in percent. The results showed that IDCSs with wider electrode fingers and lower no-touch capacitance offer higher sensitivity compared to those with narrower fingers. The sensitivity distribution analysis further revealed that the glass-embedded IDCS structure effectively maintains a balanced electric-field distribution across the sensing area, ensuring uniform and stable touch-detection performance. In future work, additional studies will be carried out to investigate other influencing parameters, such as glass thickness and permittivity of the overlay material.

## Figures and Tables

**Table 1:** Cell constant of external cells for *C_E_* calculation.

Finite Height Layer (Glass)	Infinite Height Layer (Air)
$k_{\mathrm{cell}}^{c}$ = $\frac{K\left(k^{\prime }\right)}{K\left(k\right)}$ where	$k_{\mathrm{cell}}^{c}$ = $\frac{K\left(k^{\prime }\right)}{K\left(k\right)}$ where
$k=\sqrt{\frac{t_{A}\left(t_{B}-1\right)}{t_{B}\left(t_{A}-1\right)}}$	$k=\sqrt{t_{B}+1}$
k′ = $\sqrt{1-k^{2}}$	with $t_{B}\;=\;-\frac{4\eta }{{\left(\eta \;+\;1\right)}^{2}}$
with $t_{A}={\cos h}^{2}\left(\frac{\pi \left(1+\eta \right)}{2r}\right)$	and k′ = $\sqrt{1-k^{2}}$
and $t_{B}={\cos h}^{2}\left(\frac{\pi \left(1-\eta \right)}{2r}\right)$	

**Table 2:** Cell constant of inner cells for *C_I_* calculation.

Finite Height Layer (Glass)	Infinite Height Layer (Air)
$k_{\mathrm{cell}}^{c}\;=\frac{\;K\left(k_{w}^{\prime }\right)}{\;K\left(k_{w}\right)}$ where	$k_{\mathrm{cell}}^{c}$ = $\frac{K\left(k^{\prime }\right)}{K\left(k\right)}$ where
$k_{w}=\sqrt{\frac{\left(k+1\right)\left(1-t_{B}\right)}{2\left(1-kt_{B}\right)}}$	$k=\sqrt{t_{B}+1}$
$k_{w}^{\prime }$ = $\sqrt{1-k_{w}^{2}\;}$	with $t_{B\;}=\;{\sin h}^{2}\left(j\frac{\pi \eta }{2}\right)$
where $k\;={\;\left(\frac{\vartheta_{2}\left(0,q\right)}{\vartheta_{3}\left(0,q\right)}\right)}^{2}$ with $q\;={\;e}^{-2\pi r}$	and k′ = $\sqrt{1-k^{2}}$
and $t_{B}=sn\left(K\left(k\right)\left(2\eta -1\right),m\right)\;with\;m=k^{2}$	

**Table 3:** Electrode design parameters for capacitance investigation.

Finger Gap (*F_g_*)/Finger Width (*F_w_*) /Bus Gap (*G_fb_*) (mm)			Bus Length (*L_b_*) (mm)		Finger Length (*L*) (mm)		Number of Electrode Fingers (*N*)	
1.4			35.0		37.2		14	
2.0			34.0		36.0		10	
2.4			31.2		35.2		8	
3.0			27.0		34.0		6	
***L_c_* (mm)**	***P_c_* (mm)**	***W_b_* (mm)**	***W_c_* (mm)**	**Glass thickness (mm)**		**Glass width (mm)**		**Glass length (mm)**
28	50	2	2	4		100		100

**Table 4:** Relative errors of the analytical model and simulations with/without busbars versus measurements, and busbar contribution to total capacitance (simulation only).

*F*_*w*_	Analytical Model Error (%)	Simulation Error, No Bus (%)	Simulation Error with Bus Bars (%)	Contribution of Busbars to Capacitance (%)
1.4	−2.80	3.29	6.47	3.08
2.0	−9.94	15.20	21.86	5.79
2.4	−33.89	−4.73	2.99	8.11
3.0	−43.33	−4.58	10.25	15.55

**Table 5:** Extracted *C_xyf_* versus cylinder diameter and electrode finger width after filling water.

Electrode Finger Width *F_w_* (mm)	Diameter of the Cylinder (mm)	*C_xyf_* Extracted Using Equation (12) with *C_f_* = 0 (pF)
1.4	10	0
	20	0
	30	0.01
	40	0.12
2.0	10	0
	20	0
	30	0.01
	40	0.11
2.4	10	0
	20	0.01
	30	0.04
	40	0.24
3.0	10	0
	20	0.02
	30	0.04
	40	0.36

**Table 6:** Extracted *C_xyf_* for a water-filled cylinder versus overlay-glass thickness and cylinder diameter with electrode finger width 2.0 mm.

Diameter of the Cylinder (mm)	*C_xyf_* Extracted Using Equation (12) with *C_f_* = 0 (pF)		
	3 mm Glass Overlay	4 mm Glass Overlay	6 mm Glass Overlay
10	0	0	0
20	0.01	0	0
30	0.03	0.01	0.10
40	0.28	0.11	0.09

**Table 7:** IDCS capacitance measurement in touch and no-touch state. *C_touch_* is the mean value measured as the fingertip was placed on the glass surface.

Parameter	Test Person 1	Test Person 2	Test Person 3
*F_w_* = 1.4 mm, *C_xy_* = 28.60 pF (baseline, no-touch)			
*C_touch_* (mean value)	28.10 pF	27.80 pF	27.42 pF
Standard deviation	0.034 pF	0.045 pF	0.021 pF
Intra-subject variability	0.12%	0.16%	0.08%
Inter-subject variability	Maximum relative difference of 1.24%		
*C_f_* extracted using Equation (13)	2.03 pF	3.29 pF	4.92 pF
*F_w_* = 2 mm, *C_xy_* = 17.70 pF (baseline, no-touch)			
*C_touch_* (mean value)	17.20 pF	16.80 pF	16.7 pF
Standard deviation	0.053 pF	0.051 pF	0.035 pF
Intra-subject variability	0.31%	0.30%	0.21%
Inter-subject variability	Maximum relative difference of 1.38%		
*C_f_* extracted using Equation (13)	2.06 pF	3.79 pF	4.24 pF
*F_w_* = 2.4 mm, *C_xy_* = 16.70 pF (baseline, no-touch)			
*C_touch_* (mean value)	16.00 pF	15.70 pF	15.56 pF
Standard deviation	0.048 pF	0.037 pF	0.018 pF
Intra-subject variability	0.30%	0.23%	0.12%
Inter-subject variability	Maximum relative difference of 1.28%		
*C_f_* extracted using Equation (13)	2.92 pF	4.25 pF	4.89 pF
*F_w_* = 3 mm, *C_xy_* = 12.00 pF (baseline, no-touch)			
*C_touch_* (fingertip on glass surface)	11.40 pF	11.20 pF	11.09 pF
SD	0.023 pF	0.025 pF	0.022 pF
Intra-subject variability	0.20%	0.22%	0.20%
Inter-subject variability	Maximum relative difference of 1.39%		
*C_f_* extracted using Equation (13)	2.52 pF	3.42 pF	3.94 pF

## Data Availability

The data presented in this study are available on request from the corresponding author.
